# An oxytocin-dependent social interaction between larvae and adult *C. elegans*

**DOI:** 10.1038/s41598-017-09350-7

**Published:** 2017-08-31

**Authors:** Euan Scott, Adam Hudson, Emily Feist, Fernando Calahorro, James Dillon, Raissa de Freitas, Matthew Wand, Liliane Schoofs, Vincent O’Connor, Lindy Holden-Dye

**Affiliations:** 1 Biological Sciences, Institute for Life Sciences, University of Southampton, Southampton, SO17 1BJ UK; 2 National Infection Service, Public Health England, Porton Down, Salisbury, UK; 3 Functional Genomics and Proteomics, Department of Biology, KU Leuven, Naamsestraat 59, 3000 Leuven, Belgium

## Abstract

Oxytocin has a conserved role in regulating animal social behaviour including parental-offspring interactions. Recently an oxytocin-like neuropeptide, nematocin, and its cognate receptors have been identified in the nematode *Caenorhabditis elegans*. We provide evidence for a pheromone signal produced by *C. elegans* larvae that modifies the behaviour of adult animals in an oxytocin-dependent manner increasing their probability of leaving a food patch which the larvae are populating. This increase is positively correlated to the size of the larval population but cannot be explained by food depletion nor is it modulated by biogenic amines, which suggest it is not an aversive behaviour. Moreover, the food-leaving behaviour is conspecific and pheromone dependent: *C. elegans* adults respond more strongly to *C. elegans* larvae compared to other nematode species and this effect is absent in *C. elegans daf-22* larvae which are pheromone deficient. Neurotransmitter receptors previously implicated in *C. elegans* foraging decisions NPR-1 and TYRA-3, for NPY-like neuropeptides and tyramine respectively, do not appear to be involved in oxytocin-dependent adult food-leaving. We conclude oxytocin signals within a novel neural circuit that regulates parental-offspring social behaviour in *C. elegans* and that this provides evidence for evolutionary conservation of molecular components of a parental decision making behaviour.

## Introduction

Animals have evolved intricate mechanisms that enable them to optimally locate and utilise food in their environment to satisfy their nutritional requirements, a behaviour called foraging. This is controlled by neural circuits which integrate conflicting sensory cues to drive behaviour appropriate to the specific current conditions. These cues relate not just to the abundance and quality of the food source but also the size and demographic of the population. This complexity is compounded by the need to evaluate to what extent the environment is benign or threatening. In this study, we show that the simple bacteriovorus nematode worm *Caenorhabdiditis elegans*, an exceptionally well-studied genetic model organism, makes foraging decisions which incorporate information about the presence of their well-fed offspring in the immediate environment.

Food-dependent behaviours have been extensively investigated in *C. elegans*. A paradigm that has been widely deployed involves placing a small number of adult hermaphrodite worms on a bacterial lawn of defined density and scoring the number of times individual worms leave the food patch and/or the proportion of worms that are off the food patch over a range of time-courses^1–5^. These studies have shown that adult food-leaving rate is strongly influenced by bacterial quality and density^4, 5^. Worms tend to dwell on a thick lawn of nutritional bacteria^3, 5^ but over time will increasingly leave the food patch more often and stay off the food for longer as the bacteria are consumed and the food source is depleted^3, 5, 6^. Various factors modulate the interaction of *C. elegans* with a food lawn: Pathogenic bacteria^4, 7, 8^, RNAi targeted to essential cellular processes^1^ and exposure to a range of chemical toxins^1^ all promote food-leaving. Worms fed on hard to digest bacteria^4^ or with an impaired ability to feed and digest bacteria^9^ also show enhanced food-leaving which has been interpreted as an indication of nutritional cues that regulate the behaviour^9^. The levels of metabolically important gases affects food-leaving with high carbon dioxide^10^ and oxygen levels^11^ causing worms to leave a food patch, the suggestion being that the animals integrate their response based on the benefits of feeding versus the danger of potentially toxic ambient air conditions^6^.

The assays that have been developed to investigate foraging in *C. elegans* have been coupled with genetic analyses to provide insight into the molecular substrates that underpin the worm’s decision of whether or not to leave a food patch. Some studies have taken advantage of the observation that different strains of *C. elegans* have distinct foraging behaviours. Specifically the N2 Bristol strain, the laboratory adapted wild isolate and standard reference strain, has a lower tendency to leave a bacterial lawn than the Hawaiian strain (Hw)^5^. There are striking differences in the level of food-leaving between these strains linking a plethora of genes to these behaviours^12^. Indeed, an enhanced food-leaving represents one of several sub-behaviours associated with the Hw strain^13^ in which the neuropeptide Y receptor NPR-1^2, 5, 14^ and a catecholamine receptor TYRA-3^3^ are significant determinants. Further studies have used a combination of forward and reverse genetics to unpick specific aspects of distinct cue dependent food-leaving as provoked by environment modulating cues. There is a selective role for serotonin signalling in learned avoidance of a pathogenic food source^8^ whilst neuroendocrine signalling involving TGFβ/DAF-7 and neuronal insulin signalling underpin food-leaving in response to resource depletion^2^.

In addition to being regulated by food density, quality and indicators of pathogenicity, foraging is also modified by factors relating to reproduction and fitness. Thus male *C. elegans* will leave a food patch in order to locate a mate^15^ highlighting the neural drive to reproduce can over-ride an otherwise potent nutritional cue to remain on the lawn. It has also been found that both arrested L1 or dauer larvae, which are *C. elegans* life stages generated under starvation conditions, produce signals that trigger adult food-leaving^16^ or dispersal. This is reinforced by evidence that population density can trigger dispersal for wild-type^14^ and it is also enhanced in a chitin synthase mutant, *chs-2*, which is nutritionally compromised^9^.

In this study, we provide evidence for an additional important modulator of adult *C. elegans* food-leaving behaviour, namely the specific impact of the presence of their larval progeny on their foraging response.

## Results

**Figure 1:**
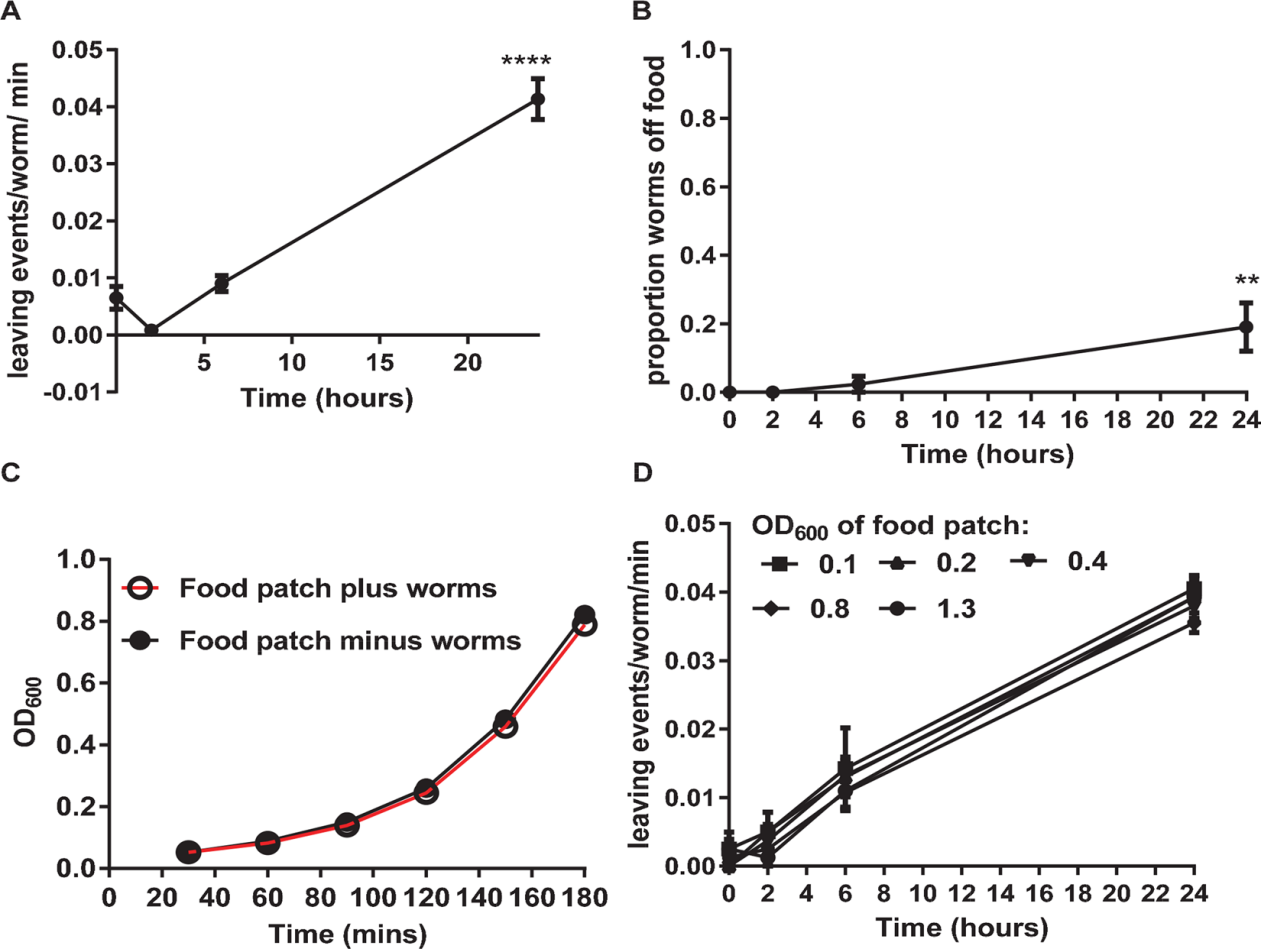
A food-leaving behaviour of adult wild-type N2 *C. elegans* that is not explained by depletion of the bacterial lawn. (**A**,**B**) Seven one day old adult wild-type (N2) *C. elegans* were placed on a defined bacterial lawn of *E. coli* OP50 and the number of leaving events scored for a period of 30 min beginning at the time points indicated. For each lawn the number of leaving events per worm was determined over the time-course and at each time point the ratio of worms off the lawn to worms on the lawn was counted. Data are mean ± s.e.mean for n = 6 lawns. One way ANOVA with Tukey’s multiple comparisons test; **P < 0.01, ****P < 0.0001. (**C**) At the end of the food-leaving assay the bacterial lawns were cut out of the agar plates and grown in LB broth at 37 °C. The growth rate of the bacterial lawns that had been exposed to worms (food patch plus worms) was compared to bacterial lawns recovered from plates cultured in an identical manner except in the absence of worms (food patch minus worms). Data are mean ± s.e.mean; n = 4. (**D**) One day old adult *C. elegans* were exposed to bacterial lawns of different optical densities and food-leaving scored as for (**A**). Data are mean ± s.e.mean for n = 4 lawns.

Previous studies have identified the tendency of *C. elegans* to transiently leave a defined ‘worm naïve’ bacterial lawn is initially very low but shows a steady increase over time such that at the later time-points the number of worms off the food patch increases^2^. We noted a similar time-dependent increase in worm leaving events and the proportion of worms off the food patch for one-day old hermaphrodites. At 2 hours there were very few leaving events over the 30 minute observation period, equivalent to less than one per worm which increased roughly 10 fold after 24 hours (Fig. 1A). This increase in the frequency of leaving events was accompanied by an increase in the proportion of worms that were distributed off the bacterial lawn at each time point (Fig. 1B). During this period the adults sustain an active feeding rate whilst they are on the bacterial lawn as observed by their high frequency of pharyngeal pumping i.e. 245 ± 3 pumps per min at 2 hours, 239 ± 4 pumps per min at 6 hours and 244 ± 2 pumps per min at 24 hours, n = 6,6, and 9 respectively. This high rate of feeding may result in the bacterial lawn becoming depleted and provide a sensory cue for food-leaving. To test whether or not there was a significant change in the density of the bacterial lawn we measured bacterial growth curves for OP50 lawns that had been cultivated for 24 hours with 7 gravid worms; that is, the conditions under which there was a progressive increase in food-leaving (Fig. 1A). These were compared to lawns incubated for 24 hours without addition of 7 worms. The growth curves for both samples were identical (Fig. 1C) suggesting that the bacterial lawn is not significantly depleted by feeding. We also tested whether or not artificially reducing the density of the bacterial lawn would impact on food-leaving and found there was no difference in the food-leaving events despite greater than 10 fold differences in optical density of the bacteria used to make the food patch (Fig. 1D). Taken together, these data indicate that depletion in the food lawn does not provide an explanation for the enhanced food-leaving observed in adult *C. elegans* over the 24 hour period.

During the time course of the food-leaving assay the adult *C. elegans*, which are gravid one day old animals, lay eggs which subsequently hatch. Typically *C. elegans* larvae take 6 to 8 hours to hatch after being laid so L1 larvae will begin to appear on the bacterial lawn between the 6 hr and 24 hour time-point. By 24 hours they will just be starting to transition to L2. Thus at the 24 hour time-point there is a mixed population containing both the original seven adults, eggs (around 200) and larvae of stages L1 and L2 (around 100). As we had no evidence to support depletion of the food source as a stimulus for enhanced food-leaving we suspected that the progressive increase in progeny of the bacterial lawn might provide a drive to enhance food-leaving.

**Figure 2:**
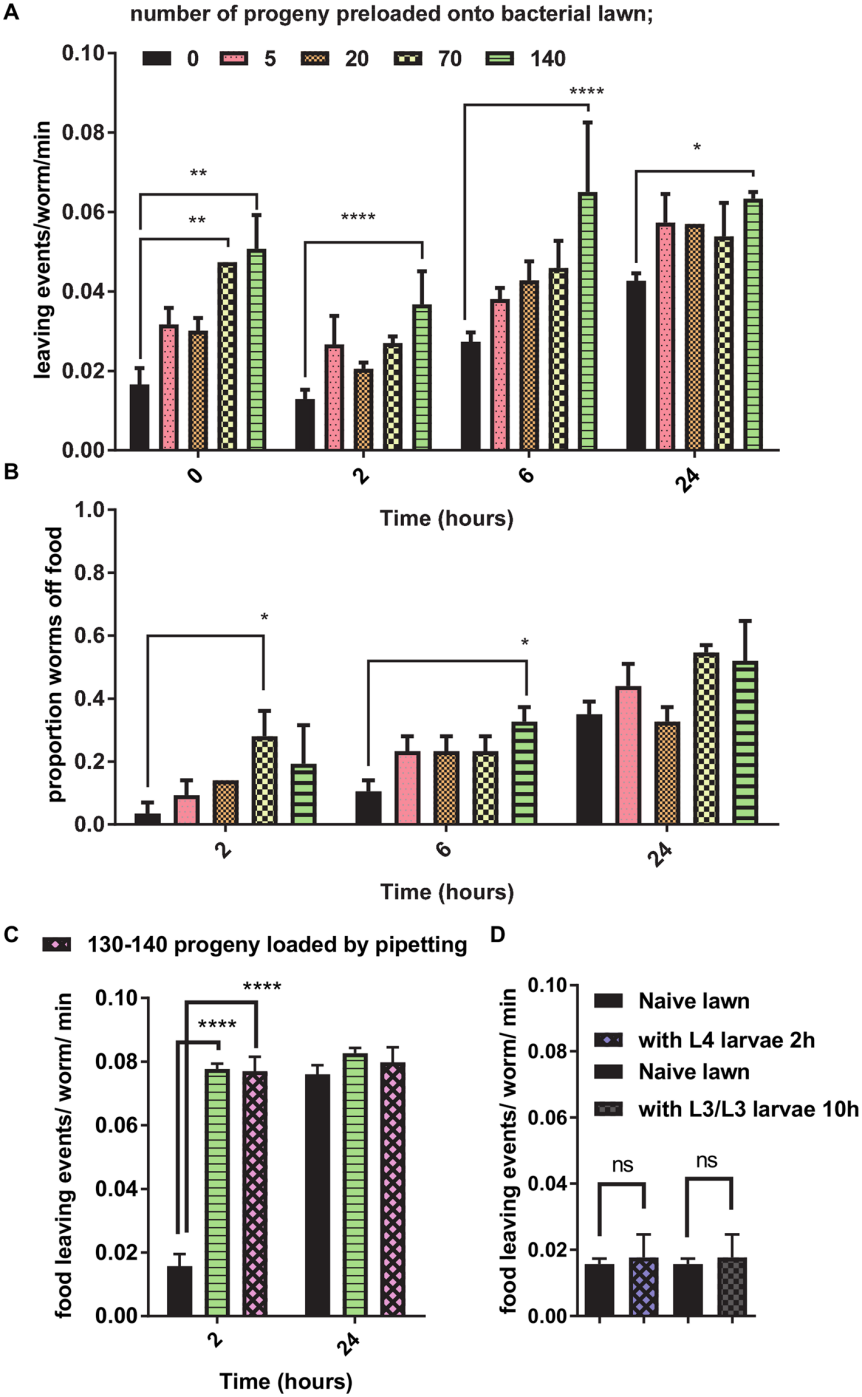
*C. elegans* L1 larvae enhance adult food-leaving. Bacterial lawns were loaded with *C. elegans* eggs at increasing density, ranging from 5 to 140, as indicated by allowing gravid adults to lays eggs on the lawn for a period of time following which the adults were removed. The eggs were left overnight to hatch into larvae and the food-leaving assay instigated by placing seven adults on each lawn. (**A**) Food-leaving and (**B**) proportion of worms off food were scored as described for Fig. 1A,B. Data are mean ± s.e.mean. ‘n’ number for treatment group ‘0’, n = 4, all other treatments n = 3. Two-way ANOVA with Tukey’s multiple comparisons test; *P < 0.05, **P < 0.01, ***P < 0.001, ****P < 0.0001. (**C**) *C. elegans* larvae enhance adult food-leaving from lawns that have never been exposed to adults. In this experiments isolated eggs were pipetted onto the lawn and the effect of the resulting larvae on adult food-leaving compared to that on plates prepared by eggs laid from gravid adults as described in A. Data are mean ± s.e.mean. ‘n’ number for ‘0’ progeny treatment group and for ‘140’ progeny loaded by the method described in A = 3, ‘n’ for progeny preloaded by pipetting = 4. Two-way ANOVA with Tukey’s multiple comparisons test; ****P < 0.0001. (**D**) L4 larvae did not enhance adult food-leaving. Bacterial lawns were conditioned with 120 L4s for 2 hours after which adult food-leaving was scored. Data are mean ± s.e.mean. ‘n’ = 3 for each experimental group. p > 0.05 unpaired Student’s t-test. This experiment was repeated to allow for longer pre-conditioning of the lawn by picking L3s onto the lawn and leaving them for 10 hours by which time the larvae had all developed into L4s. The leaving rate of adults (picked onto the lawn 2 hours before) was scored. Data are mean ± s.e.mean. ‘n’ = 5 for each experimental group. p > 0.05 unpaired Student’s t-test.

To test our hypothesis we placed one day old hermaphrodite *C. elegans* on food patches that had been pre-loaded with increasing numbers of eggs (between 0 and 140) the previous day and which had developed into larvae. Remarkably, adult *C. elegans* placed on bacterial lawns that had been populated with 140 progeny (L1 larvae) showed an immediate high rate of food-leaving, similar to the food-leaving rate of worms placed on bacterial lawns without progeny after 24 hours (Figs 1A and 2A). Furthermore, this had the appearance of dose-dependency with a threshold of between 20 and 70 progeny (Fig. 2A). Additionally, the food-leaving of the adult worms placed on the lawns pre-loaded with the progeny increased slightly after 24 hours compared to adult worms placed on lawns that had not been pre-loaded with progeny, presumably because their own progeny populate the plate and further serve to increase the number of larvae on the lawn (Fig. 2A). However, the relative small increase between the experimental groups, control and pre-loaded with 140 progeny at the 24 hour time point suggests that there may be a plateauing effect with it reaching a near maximal level in the presence of 140 plus progeny. For plates preloaded with progeny the increase in food-leaving was accompanied by an increase in the proportion of worms off food (Fig. 2B). To further test whether or not the cue for adult food-leaving is offspring derived, instead of an enduring signal permeating the lawn left by the adults that were used to preload the lawns with eggs prior to their removal, we used another method to load the plates with progeny. For this, we isolated *C. elegans* eggs from gravid adults and pipetted them onto the bacterial lawn. We found that adult food-leaving on lawns preloaded with progeny in this manner was the same as that for lawns preloaded by allowing gravid adults to lay eggs before their removal (Fig. 2C).

**Figure 3:**
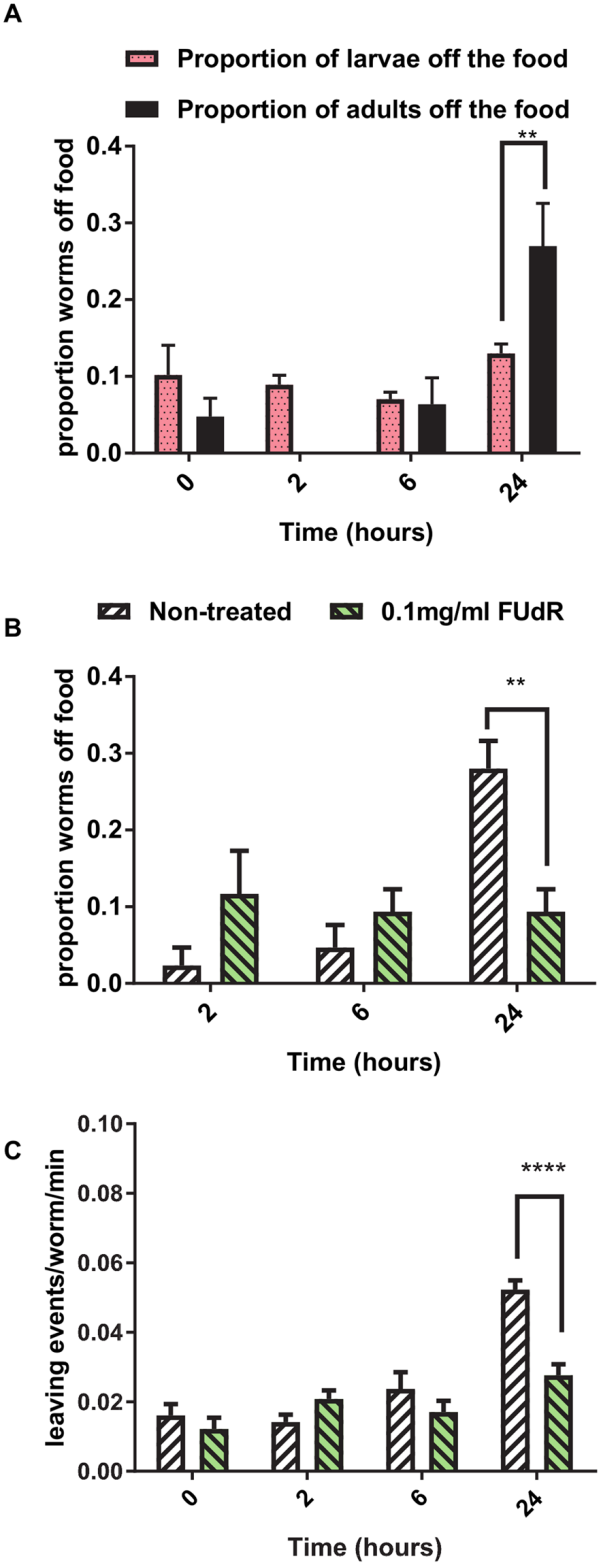
The food-leaving response is not seen in larvae nor in sterile worms. (**A**) The proportion of worms off food were scored as described for Fig. 1B except that in these assays both adult worms and larvae were scored in parallel. n = 4 bacterial lawns. Data are mean ± s.e.mean. One way ANOVA with Bonferroni multiple comparisons. (**B**,**C**) *C. elegans* were pre-treated with 0.1 mg/ml FUdR to induce sterilisation. These worms lay eggs that do not hatch. They were subjected to the food-leaving assay as described in Fig. 1. Control worms were treated in an identical manner except for the omission of FUdR. Food-leaving and the proportion of worms off food was scored as described in Fig. 1. Data are mean ± s.e.mean; n = 5 lawns for both treatment groups. Two way ANOVA with Bonferroni multiple comparisons. *P < 0.05, **P < 0.01, ***P < 0.001, ****P < 0.0001.

This suggests that a cue from the L1 larvae, rather than from the adults that supplied the eggs for preloading the plates, drives the enhanced food-leaving response in adults. The selective effect of early stage larvae on adult food-leaving is further reinforced by an experiment in which bacterial lawns were preloaded with 120 L4 larvae and then the impact on the food-leaving of adults was observed. There was no significant enhancement of food-leaving after L4 larvae had populated the lawn for 2 hours (Fig. 2D). In order to check whether an extended time of exposure of the lawn to later stage larvae might drive adult food-leaving the lawns were populated with 140 L3s which were allowed to inhabit the lawn for 10 hours before the food-leaving rate of adults, placed on the lawn 2 hours previously, was scored. By this time, all the L3s had developed into L4s and, as with the shorter time of exposure, an increase in adult food-leaving was not observed (Fig. 2D). This suggests that pre-conditioning the lawn with L1 larvae is required to drive the adult food-leaving rate. Furthermore, whilst adult worms exhibited enhanced food-leaving this behaviour was not observed in the larvae themselves suggesting that the response is specific for the adults (Fig. 3A).

To investigate the possibility that early stage larvae, not the eggs produced by adult *C. elegans*, enhanced adult food-leaving we sterilised young adult worms by pre-treating them with the DNA synthesis inhibitor 0.1 mg/ml 5-fluoro-2′-deoxyuridine (FUdR)^17, 18^. The FUdR treated worms laid eggs that did not hatch and they failed to show enhanced food-leaving over time (Fig. 3B,C). This indicates that it is the L1 larvae that are largely responsible for the enhanced food-leaving effect. Altogether, these results show that *C. elegans* L1 larvae provide a significant drive to enhance the food-leaving behaviour of adults.

**Figure 4:**
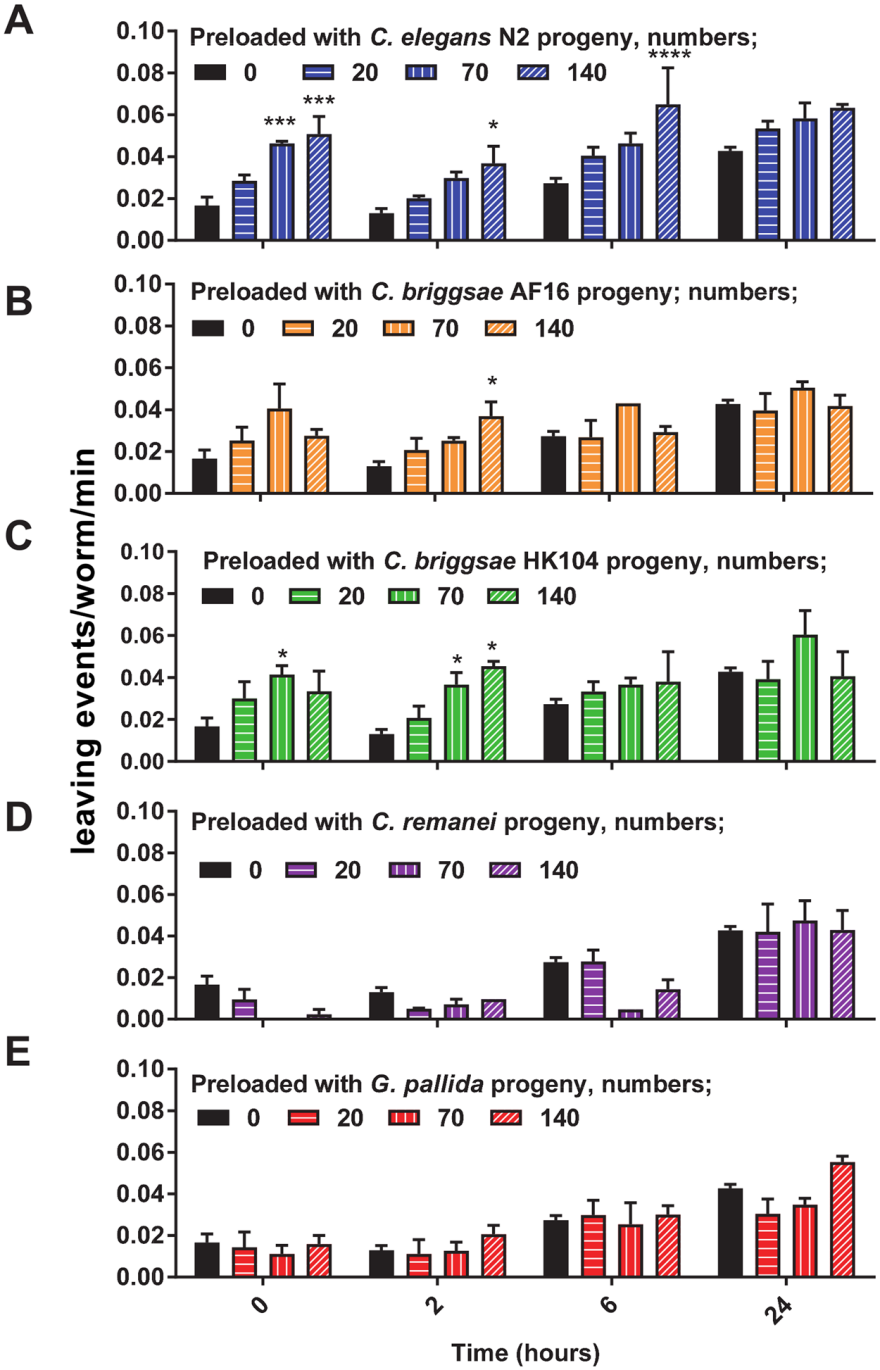
Progeny enhanced food-leaving response in adult *C. elegans* is conspecific. Different numbers of larvae, as indicated, from *C. elegans*, *C. briggsae, C. remanei* and *G. pallida* were pre-loaded onto bacterial lawns before adult *C. elegans* were added and assayed for food-leaving as described in Fig. 1A. Data are mean ± s.e.mean. n = 3 lawns for each experimental group. Significant difference is shown with respect to the no treatment group for each time-point. Two way ANOVA with Bonferroni multiple comparisons. *P < 0.05, **P < 0.01, **P < 0.001, ****P < 0.0001.

As our data indicated that adult *C. elegans* will increasingly leave a food source that is populated by predominantly L1 larvae in the absence of any obvious depletion in the quantity of food we next considered whether or not deterioration of the quality of the food might provide an explanation for the behaviour. We hypothesised that if excretory products from the larvae populating the food promote food-leaving then the same response should be observed in adult *C. elegans* regardless of the species of larvae used to pre-load the bacterial lawn. Therefore, we tested *Caenorhabditis briggsae* strains AF16 and HK104 which are wild isolates of a hermaphroditic relation of *C. elegans* that shares habitats with *C. elegans* ^19, 20^ and *Caenorhabditis remanei*, JU724. This latter wild isolate, like *C. elegans*, is found in fermenting environments^20^. We also tested J2 juveniles of *Globodera pallida*. *G. pallida* is a plant parasitic nematode that infects and proliferates inside potato roots, and unlike the three *Caenorhabditis* species is not a bacteriovore^21^. As before, the presence of N2 larvae increased the food-leaving of N2 adults (Fig. 4A–D) as indicated by the immediate increase in leaving rate when the adults were placed on the lawns with the progeny. In contrast only a weak enhancement of food-leaving was observed for *C. briggsae* larvae (Fig. 4A,B) whilst for *C. remanei* (Fig. 4C) and *G. pallida* (Fig. 4D) there was no significant effect. Thus, the ability of larvae to drive the adult food-leaving response in *C. elegans* would appear to be conspecific and not due to a reduction in either the quantity, nor in the quality, of the food.

**Figure 5:**
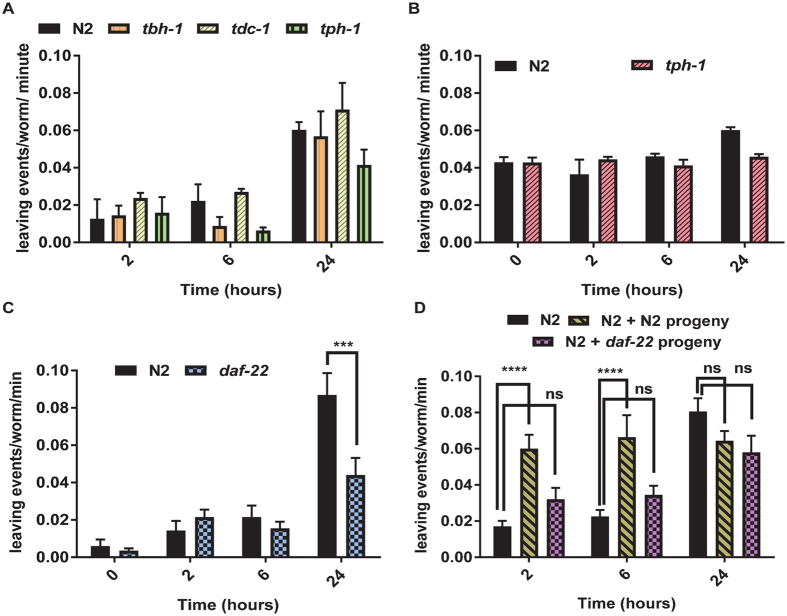
The progeny induced food-leaving response in adult *C. elegans* is not dependent on serotonin nor octopamine, but does require *daf-22* pheromone signalling. (**A**) Food-leaving for seven wild-type N2, *tbh-1 (n3247)*, *tdc-1 (n3419)* or *tph-1(n4622)* adults placed on bacterial lawns. (**B**) Food-leaving for seven wild-type N2 or *tph-1(n4622)* adults placed on bacterial lawns preloaded with 140 wild-type larvae. (**C**) Food-leaving was scored, as described in Fig. 1A, for wild-type and the pheromone deficient *daf-22 (m130)* mutant. n = 4 bacterial lawns. (**D**) Food-leaving for wild-type N2 worms in the presence of either wild-type larvae or *daf-22* larvae. N2 adults were placed on bacterial lawns without pre-loaded larvae (n = 7) or with 130 N2 larvae (n = 5) or 130 *daf-22* (n = 4) larvae. Data are mean ± s.e.mean. Two way ANOVA with Bonferonni multiple comparisons. **P < 0.01, ***P < 0.001, ****P < 0.0001.

Whilst the adult food-leaving behaviour did not appear to be explained by deterioration of the food source, we were nonetheless interested to investigate whether or not it has any of the characteristics of an aversive response. Biogenic amines, and in particular serotonin, are key regulators of the interaction of *C. elegans* with its food and have been implicated in avoidance of pathogenic food^8, 22^ and dwelling states on food^23^. Therefore, we tested mutants for biogenic amines, *tdc-1* and *tbh-1* which are deficient in tyramine and octopamine^24, 25^ and *tph-1* which is lacking serotonin^26^. Mutants for *tdc-1* and *tbh-1* showed the same food-leaving as wild-type adults (Fig. 5A) therefore tyramine and octopamine are not involved. There was a slight reduction in food-leaving in *tph-1* therefore we re-tested this mutant in the format of the progeny enhanced food-leaving assay and showed that it behaved in the same way as N2 adults (Fig. 5B). This reinforces the suggestion that the progeny enhanced food-leaving in adults is not an aversive response to poor quality food as serotonin is an important regulator of aversive behaviour^8, 22^.

By extrapolation, the data showing that food depletion and deterioration do not trigger adult food-leaving in the presence of larvae, invites an alternative explanation in which a pheromone signal from the larvae increases the frequency of food-leaving in the adults. In support of this we found that *daf-22* mutants^27^, which are deficient in pheromone production, did not exhibit food-leaving (Fig. 5C). To test whether or not the deficit in the behaviour can be ascribed to a loss of signal from *daf-22* larvae to the adults we tested the food-leaving rate of N2 adults on bacterial lawns that had been preloaded with either N2 or *daf-22* progeny. Food-leaving was elicited to a significantly lesser extent by *daf-22* larvae, supporting the idea that a *daf-22* dependent signal from the larvae elicits food-leaving behaviour in adults.

**Figure 6:**
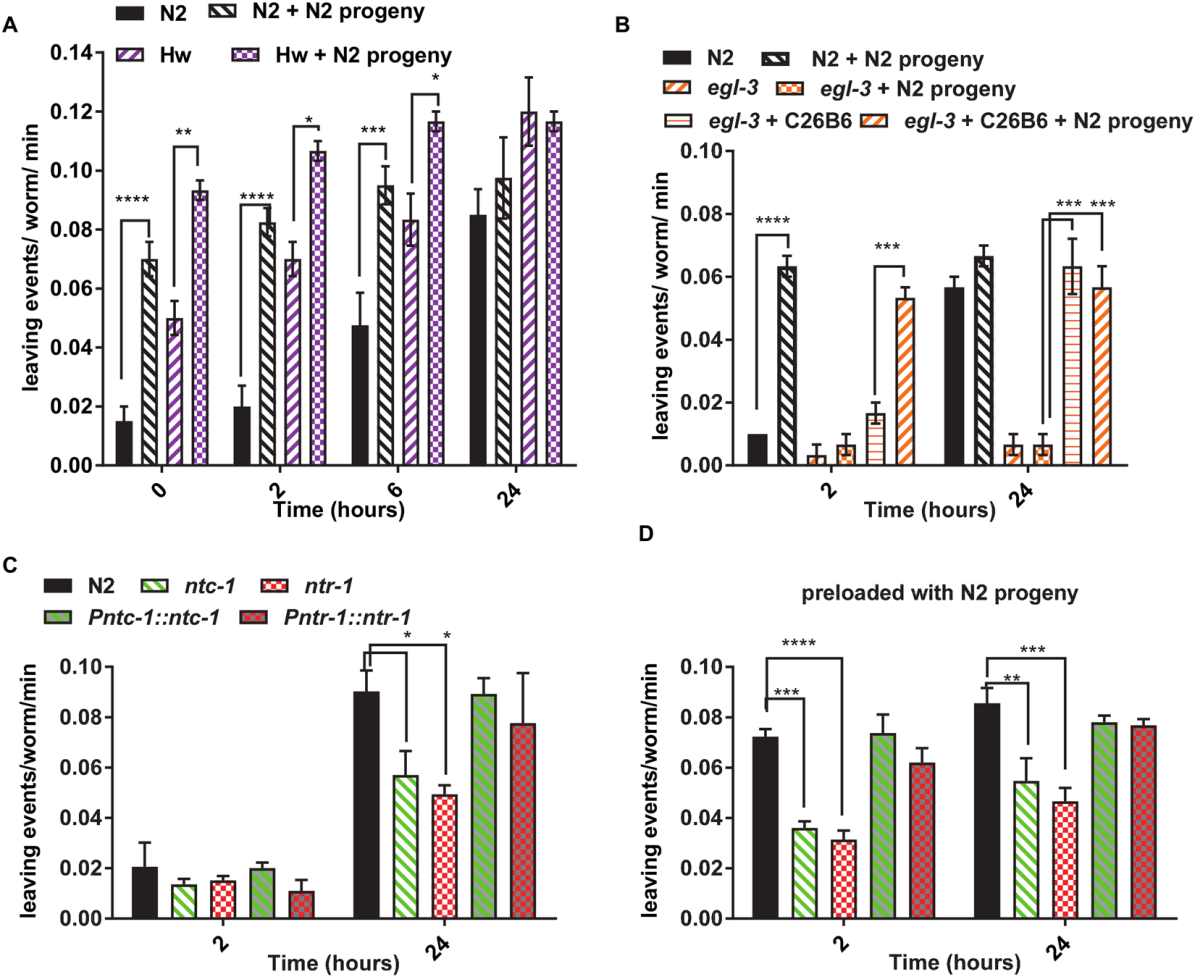
Progeny enhanced food-leaving is observed in the Hawaiian (Hw) strain of *C. elegans* but not in the neuropeptide deficient mutant *egl-3* or in nematocin signalling mutants. (**A**) Food-leaving was scored for wild-type N2 adults and Hawaiian strain as described in Fig. 1A. In the absence and presence of 140 wild-type N2 larvae. n = 4 for N2 and n = 3 for Hw. (**B**) Food-leaving was compared between wild-type N2, *egl-3* and transgenic *egl-3* mutants expressing the cosmid C26B6 which harbours genomic sequence for *egl-3*. For this assay each strain tested was assayed in the absence or presence of 140 *C. elegans* larvae as indicated. n = 3 lawns for each experimental group. (**C**) Food-leaving was scored for wild-type N2 adults and nematocin mutants as described in Fig. 1A. N2 n = 3; *ntc-1(tm2385)(*LSC42*)* n = 5; *ntr-1(tm2765)*(LSC48) n = 5; *Pntc-1::ntc-1*(LSC455) n = 4; *Pntr-1::ntr-1* (LSC402) n = 3. (**D**) The comparison between wild-type N2, nematocin mutants and rescue lines was repeated on bacterial lawns pre-loaded with 140 N2 progeny. N2 n = 4; *ntc-1(tm2385)*(LSC42) n = 6; *ntr-1(tm2765)*(LSC48) n = 5; *Pntc-1::ntc-1*(LSC455) n = 4; *Pntr-1::ntr-1* (LSC402) n = 4. Data are expressed as mean ± s.e.mean. Two way ANOVA with Dunnett’s multiple comparisons test. *P < 0.05, **P < 0.01, ***P < 0.001, ****P < 0.0001.

To define further molecular determinants of progeny enhanced food-leaving we made use of the Hawaiian strain^13^. Its increased tendency to leave a bacterial lawn^5, 14^ has provided a route to Quantitative Trait Loci analysis (QTL) to identify genetic determinants of this polygenic behaviour^3, 12^. Interestingly, the base-line for the Hw food-leaving response was elevated compared to N2 across all the time-points: Previous analyses of the increased food-leaving of Hw has suggested that this may at least in part be explained by increased motility of the Hw strain compared to N2^3^. Notably however the progeny enhancement was superimposed on this raised overall food-leaving behaviour at each of the time-points (Fig. 6A). Therefore, the genetic determinants of the increased food-leaving of Hw, major players in which are NPR-1 and TYRA-3^3, 5^, does not occlude the progeny enhancement. This suggests that the progeny enhanced food-leaving has revealed a new and distinct neural circuit involved in complex decision making in *C. elegans* adults.

Given that neuropeptides are well recognised modulators of behavioural plasticity^28^ we made use of a well-established approach for testing for neuropeptide involvement in the food-leaving behaviour using the mutant *egl-3(ok979*). This provides a global reduction in neuropeptide content as it is deficient in a proprotein convertase needed for processing of numerous neuropeptides in *C. elegans* ^29, 30^. We found that *egl-3* worms were deficient in the enhanced food-leaving response and this was rescued by expression of a wild-type copy of *egl-3* following cosmid injection (Fig. 6B). This is consistent with a role for neuropeptide signalling in *C. elegans* as a major determinant of the food-leaving response although this could be an indirect consequence of an effect on locomotory behaviour: Whilst measurements of *egl-3* speed and posture are not significantly different from wild-type^31^ this mutant is noted for its tendency to coil^32^ and we cannot rule out that this may impair its ability to leave the food lawn.

Nonetheless, given this indication for an involvement of neuropeptide signalling, we speculated that nematocin, the *C. elegans* homologue of the mammalian peptide hormone oxytocin^33^, may underpin the progeny enhanced food-leaving response. In mammals oxytocin is an important regulator of social behaviours, including parental bonding^34^. Nematocin has been shown to control mate searching and mating behaviours in male *C. elegans*, as well as gustatory learning in the form of salt chemotaxis^35, 36^. Moreover, unlike *egl-3* mutants, no movement deficits have been reported for nematocin signalling mutants^36^. We tested *C. elegans* deficient in both the nematocin peptide (*ntc-1*(tm2385)) and its two receptors, *ntr-1(tm2765)* and *ntr-2 (tm2243)*, in the food-leaving assay. We first investigated the reproductive capacity of these strains by counting the number of progeny produced in 24 hours by seven one day old adults. This revealed a significant reduction for *ntc-1*, *ntr-1* and *ntr-2* (Table 1). As they show this reproductive defect which might confound interpretation of a progeny enhanced food-leaving response we tested the effect of progeny induced food-leaving in the nematocin mutants by pre-loading the bacterial lawns with N2 larvae, as before, and then compared the food-leaving of adult wild-type and the nematocin signalling mutants. This revealed that nematocin mutant adults are deficient in progeny enhanced food-leaving (N2 0.04158 ± 0.002250; *ntc-1* 0.0190 ± 0.001905; *ntr-1* 0.02056 ± 0.006390; *ntr-2* 0.02056 ± 0.001556; food-leaving events per worm per minute; n = 4,4,3 and 3 respectively; p < 0.01 compared to N2 for *ntc-1*, *ntr-1* and *ntr-2*; one way ANOVA with Bonferroni multiple comparisons). To confirm this we repeated the assay in outcrossed and rescue strains for *ntc-1* and *ntr-1*. Mutants for *ntc-1* and *ntr-1* both showed reduced food-leaving compared to N2 and this was rescued by expression of *ntc-1* or *ntr-1*, respectively from their native promoters (Fig. 6C,D).

**Table 1:** The reproductive capacity of nematocin signalling mutants. The larvae produced by seven one day old adults in a 24 hour period was scored. LSC42 and LSC48 were outcrossed 3x and 4x, respectively. FX02243 was not outcrossed. Data are mean ± s.e.mean. One way ANOVA with Bonferroni multiple comparisons. *p < 0.05 and **p < 0.01 with respect to N2.

GENOTYPE	Strain	larvae produced
	N2	128 ± 11 (5)
*ntc-1(tm2385)*	LSC42	92 ± 6 (5)*
*ntr-1(tm2765)*	LSC48	79 ± 7 (5)**
*ntr-2(tm2243)*	FX02243	49 ± 21 (3)**

Therefore nematocin signalling in adult worms mediates a *daf-22* dependent signal emanating from their larvae and drives the adults to leave the food patch with increasing frequency.

## Discussion

Measuring food-leaving behaviour in *C. elegans* is a binary assay that provides phenotypic quantification of a simple behavioural choice, whether to stay on a bacterial food source or to leave it^3^. To execute a food-leaving event the worm is driven by sensory modalities in the locality of its food; integration of these leads to a shift in their motor program such that they leave the food patch. Studies on the genetics of *C. elegans* have identified cellular control within defined microcircuits that integrate environmental cues and drive the outcome which is a food-leaving response^2^. Overall, this highlights that the simple measurement of the worms’ tendency to remain on﻿, or leave, a food patch represents a powerful route to investigate molecular, cellular and microcircuit control of complex behaviour.

In this study we characterised the time-dependence of food-leaving by N2 adults over 24 hours and noted the previously observed enhanced dispersal from a food patch in the relatively benign environment of an *E. coli* OP50 lawn^2, 5, 14^. Our experiments used N2 worms and dense OP50 bacterial lawns to provide conditions that converge to ensure a relatively low rate of initial food-leaving. Indeed the initial rate of leaving from a lawn of 50 µl of OP50(OD_600_ 0.8) was in the region of 0.02 leaving events/worm/minute which is comparable to the leaving rate previously reported for the same number of N2s on a lawn of 10 µl of HB101 (Ab_600nm_ 2.0) in the region of 0.01 leaving events/worm/minute^3^. By pre-loading the bacterial lawns with progeny (L1 larvae), and testing sterile adults, we have shown that the increase in population of larvae drives food-leaving specifically in adults.

The adult food-leaving that is driven by the worm’s progeny is distinct from a previously described food-leaving behaviour driven by nutritional deprivation^9^: In our assays the adults and the larvae were well fed and the assays were conducted in the presence of abundant food. It is also distinct in terms of the magnitude of effect, which is greater in nutritionally deprived worms. This argues for discrete modulation of adult foraging decisions by the immediate proximity of their progeny on the food patch.

We have investigated a number of possible explanations for progeny enhanced adult food-leaving. In particular, we considered whether or not the negative impact of the increase in population density on either food quantity or quality has a role. Our measurements of the growth curves of bacterial lawns conditioned for 24 hours with or without worms did not reveal any indication of a significant depletion of the food during the assay. Whilst this on its own does not negate the possibility that there is an undetectable change in food quantity or quality, we argue that such a change is unlikely to provide an explanation for progeny enhanced food-leaving in adult *C. elegans*: If this were the case one might expect to see the same food-leaving response regardless of the species of nematode progeny that were used to pre-condition the bacterial lawn. The conspecific nature of the food-leaving behaviour in adult *C. elegans* in response to progeny of their own species, but not in response to other nematode species, argues that this is not an indirect consequence of depletion or deterioration of the food lawn. Furthermore, our observation that this behaviour is not modified by serotonin signalling, a known regulator of aversive behaviour^1, 8^, provides further argument that the response does not arise because the presence of the larvae modifies the bacteria making the lawn aversive to the adults.

Progeny enhanced food-leaving could be interpreted as a parental response in the adults to the increasing population density. Arguably, this would be beneficial to the larvae allowing them to take full advantage of the food source on which they hatched. Our data suggest a signal is transmitted from the larvae to the adults on the bacterial lawn to induce them to leave the food patch. An important class of molecules are the ascarosides, which act to control numerous behaviours^37^. One of these behaviours is entry to and exit from the dauer stage in the *C. elegans* lifecycle in response to varying food and population levels, as part of the ‘dauer pheromone’^38^. Other behaviours in *C. elegans* controlled by ascarosides include regulating mating behaviour^39, 40^, modifying olfactory preferences^38–40^ and dispersal^16^. The behaviours that ascarosides control have been shown to vary widely depending on the chemical compositions of the ascaroside mixture as well as the stage of the *C. elegans* lifecycle when the ascarosides are produced^16, 41, 42^ and varies for different natural isolates of *C. elegans* ^43^. There is also evidence for an ascaroside independent signal that promotes survival of L1 larvae subjected to starvation^44^. Similar ascaroside and non-ascaroside cues may be expected and differentially expressed during the hatching and development of progeny. We found that the pheromone deficient mutant *daf-22* does not show enhanced food-leaving consistent with the idea that an ascaroside signal from *C. elegans* larvae enhances food-leaving in adults as part of a parental behavioural response. This further distinguishes progeny enhanced food-leaving from that observed in nutritionally deprived worms as the latter is not *daf-22* dependent^9^. The experimental paradigm we have established for progeny enhanced food-leaving will provide a tractable platform for resolving further chemical cues underpinning conspecific interactions.

Intriguingly, the progeny enhanced food-leaving we have described is independent of the neural circuit that has been previously described to regulate foraging decisions in the Hw strain^3^. Rather it engages a nematocin signal and its cognate receptors NTR-1 and possibly, in addition, NTR-2^35, 36^. This oxytocin/vasopressin like peptide signalling pathway^33, 45^ is important for parental care and pair bonding in mammals^46^ and has an evolutionary conserved role in reproductive related behaviours^47^. We found that nematocin signalling is required in the adults for them to engage the progeny induced food-leaving behaviour. Given that the null nematocin hermaphrodites have normal locomotion speed^36^ and chemotaxis^35^ it is unlikely that this deficit is due to an indirect effect on a sub-behaviour required for the response. Rather it suggests that nematocin is required in circuits that integrate a chemical cue from the larvae in the context of the food source to drive dispersal in the adults. Oxytocin signalling is also recognised for its intimate role in social interactions in general and therefore it is possible that nematocin signalling between adults could be involved in population density effects previously reported for food-leaving behaviours^9^. Nematocin and its receptors are quite broadly expressed in *C. elegans*, in sensory neurones, interneurones and motorneurones^35, 36^. This places the signals in neural circuits that are involved in detecting and responding to environmental cues. It will be interesting to understand how the signalling is organised and to what extent it deploys neurohormonal versus local transmission compared to mammalian oxytocin signalling^48, 49^.

In conclusion, our data show that well fed early stage larvae generate potent inter-organismal signalling. This is in addition to the previously reported signalling that emanates from starved larvae^44^. This signal, which may reflect differential ascaroside activity exhibits a dose-dependent modulation of food-leaving activity. Previous determinants implicated in food-leaving were not attributed to this context^2^. Our observation, that the behaviour is dependent on intact nematocin signalling, points to a novel neural circuit mediating an offspring-dependent social interaction in *C. elegans*.

## Materials and Methods

All *Caenorhabditis* strains were maintained on 5 cm Nematode Growth Media (NGM) plates, according to standard methods^50^. Strains used were *C. elegans* Bristol N2; Hawaiian strain CB4856; MT14984 *tph-1 (n4622)*; MT13113 *tdc-1 (n3419)*; MT9455 *tbh-1 (n3247)*; XA3441 *egl-3 (ok979)*; FX02385 *ntc-1(tm2385);* DR476 *daf-22 (m130)*; FX02765 *ntr-1(tm2765)* and FX02243 *ntr-2 (tm2243) C. briggsae* HK104 and AF16, *C. remanei* JU724. The *egl-3* rescue line was generated from XA3441 by microinjection of 10ng/µl of cosmid C26B6 together with the transformation marker 50ng/µl *pmyo-2::gfp* as previously described^31^. *Gfp* expressing worms were selected for analysis. Animals were synchronised prior to assay by being picked at the L4 larval stage and developed for 16 hours (or overnight) prior to examination. The outcrossed strains for *ntc-1* and *ntr-1* mutants were LSC42 and LSC48, respectively. Rescue constructs for the nematocin receptor (*ntr-1*) and nematocin precursor (*ntc-1*) were made using the pSM SL2 GFP vector (kindly provided by C. Bargmann, Rockefeller University, New York, USA). *ntc-1* genomic DNA or *ntr-1* cDNA was cloned between the SalI and KpnI sites of the pSM vector, while the corresponding promoters (3.6 kb or 4 kb of sequence upstream of the *ntc-1* or *ntr-1* start codon, respectively) were cloned between the FseI and AscI sites. Microinjection of these plasmids into LSC48 or LSC42 yielded the rescue strains LSC402: LSC48 *lstEx326* [*Pntr-1::ntr-1(tm2765):: SL2 gfp 100ng/ul; Pelt-2::gfp*] and LSC455: LSC42 *lstEx374* [*Pntc-1(tm2385)::ntc-1:: SL2 gfp 50ng/ul; Pelt-2::gfp*], respectively.

For experiments using *Globodera pallida* free living J2 stage nematodes were collected from hatchings of infected roots. This was done by incubating potato root cysts in individual wells in a 3:1 mix of double distilled H_2_O and potato root diffusate. J2 stage animals that emerged within a 48 hour window were collected from these hatchings, washed with distilled water and known numbers pipetted onto OP50 lawns. These J2s were left to dry before the adult *C. elegans* to be assayed were introduced onto the plate.

Cultures of *E. coli* OP50 were maintained on 9 cm LB plates. For seeding *C. elegans* NGM plates, individual bacterial colonies where grown in LB at 37 °C overnight in a rotary incubator before being diluted 1 in 100 and grown at 37 °C in LB until an OD_600_ of 0.8 was reached. NGM plates were prepared according to standard protocols^50^, stored at room temperature (20 °C) and used within 5 days of pouring. For each paired food-leaving assay plates for the control and experimental groups were taken from the same batch. NGM plates were prepared with a bacterial lawn as follows: Upon reaching an OD_600_ of 0.8, 50 µl of OP50 (which is equivalent to 4 × 10^7^ colony forming units) was pipetted onto 5 cm NGM plates then left to grow overnight (18 hours) at 20 °C to form a bacterial lawn, after which these were used as food-leaving assay plates. The plates used for the serial dilution of OP50 experiments were set up in the same way with the exception that a range of dilutions of OP50, as indicated, was pipetted onto the agar surface.

To test OP50 growth curves from bacterial lawns with or without *C. elegans*, we removed the adult *C. elegans* from the worm cultivated lawns which were subject to the conditions under which there was a progressive increase in food-leaving (Fig. 1A). Under sterile conditions, we cut out the OP50 patches from these lawns. These were directly compared to OP50 lawns generated from the same OD_600_ 0.8 OP50 but incubated for 24 hours without addition of seven worms.

These samples were grown in 3 ml LB at 37 °C under sterile conditions with aeration for three hours. The optical density of each culture was measured every 30 mins for the 3 hours to estimate the relative growth curves.

*C. elegans* were age synchronised by picking L4 onto culture plates the day before the experiments. On the day of the food-leaving assay, seven one day old *C. elegans* hermaphrodites of each strain under investigation were picked from these plates onto the middle of the OP50 lawn. Once the worms had been placed on the plate, they were allowed 10 minutes to recover from picking before commencing the food-leaving measurements. Food-leaving was scored by visual observation using a Nikon SMZ800 binocular zoom microscope at ×10 magnification. A leaving event was defined as the whole body of one *C. elegans* completely leaving the food patch. The number of food-leaving events was recorded over 30 minutes at time 0 (10 min after the transfer of the worms to the lawn) and at time points 2, 6 and 24 hours as indicated. In addition to this dynamic measurement the proportion of the seven adult animals off the food patch was recorded at each of these same time points. For some experiments, as indicated, the number of eggs and larvae on the plate after 24 hours was counted. In addition, in some assays pharyngeal pumping was measured by visual observation of movements of the terminal bulb grinder as previously described^51^.

To examine how progeny produced during the 24 hour time course influences food-leaving bacterial lawns were laced with eggs before adding the adult worms. Assay plates were prepared as above with the modification that both control plates and plates to be preloaded with eggs before the food-leaving assay were seeded with OP50 two days before the experiment. This protocol was adopted to normalise the bacterial growth of the control and the progeny laced lawn to an extra 24 hours pre-assay growth. Gravid adults were placed on bacterial lawns and left to lay a defined numbers of eggs on the food patch before being removed. The eggs were then left overnight to hatch into larvae. The number of eggs placed on each plate ranged from 5 up to 150. The highest density value was chosen as this is equivalent to the number of larvae that would be present on each lawn after it had been populated by seven adult worms for 24 hours. The next day, approximately, 18 hours after removing the adults, a food-leaving assay was performed as above, measuring the food-leaving behaviour of adult *C. elegans* subsequently added to the plates. The experiment was repeated by varying indicated numbers of *C. briggsae* and *C. remanei* larvae. For *G. pallida* juveniles, defined numbers of hatched J2s were added directly to the plates prior to addition of adult *C. elegans*. As an alternative approach to pre-loading lawns with progeny avoiding the need to expose the lawns to gravid adults which might leave a pheromone trace we pipetted isolated eggs directly onto the bacterial lawns. Isolated eggs were prepared from gravid adults by washing them off plates in 1 ml M9 into an Eppendorf containing 500 µl bleaching solution (20% bleach, 25% 1 M NaOH 55% water). The tube was left for 5 minutes and was then pelleted by centrifuging at 1500 rpm for 2 minutes. The supernatant was removed and replaced with 100 µl M9. 25 µl of this solution was pipetted onto the food lawn and eggs were left to hatch resulting in 130–140 L1 larvae the following day.

To test the effect of L4 larvae on adult food-leaving 120 L3s or L4s were picked directly onto a bacterial lawn and left to settle for nine hours or one hour, respectively. At this time-point seven adults were picked onto the lawn to initiate the food-leaving assay. Food-leaving was scored after one hour.

To test the effect of sterility﻿,﻿ NGM plates were prepared as above and were seeded with OP50. The day before the assay, 5-fluoro-2′-deoxyuridine (FUdR) (Sigma) diluted in distilled water was pipetted onto the NGM plates to a final concentration of 0.1 mg/ml. This method was performed in order to not affect the bacterial lawn, as adding FUdR to molten agar affects bacteria’s ability to grow on NGM plates^18, 52^. The following day, adults were added to the plates and the food-leaving assay was performed as indicated above.

Data are presented as the mean ± s.e.mean for ‘n’ experiments. For food-leaving assays each ‘n’ represents one bacterial lawn with seven adults. Statistically significant differences between experimental groups were analysed using GraphPad Prism software (version 6, San Diego). One way or two-way ANOVA was used as appropriate and post-hoc tests. Significance was set at p < 0.05.
